# Pastoral Practices of Ethical Negotiation: Community Nurses and Implementation of Patient Self‐Management

**DOI:** 10.1111/1467-9566.70095

**Published:** 2025-10-02

**Authors:** Hannah Kendrick

**Affiliations:** ^1^ Care Policy and Evaluation Centre Department of Health Policy London School of Economics and Political Science London UK

**Keywords:** community health teams, community nurses, Foucault, governmentality, pastoral power, professional identity, self‐management, UK

## Abstract

Health policy that aims for patients to take greater responsibility for self‐managing their long‐term condition has had increasing global significance. Sociologists, drawing on Foucault's governmentality theory, have explored the way in which ‘responsibilised’ identities are constructed for patients, encouraging them to make well‐informed and self‐sufficient decisions about their health, as well as how health professionals, acting as ‘pastors’, attempt to shape these desirable patient subjectivities. This paper draws on ethnographic data collected within a community‐based integrated care (CBIC) service in England to explore how community nurses were encouraged to seek out and eliminate ‘waste’ by discharging patients to self‐management. Building on Waring and Martin’s model of pastoral practices, this paper demonstrates how nurses engaged in pastoral ‘practices of ethical negotiation’ when trying to reconcile sometimes competing discourses of ‘waste watching’ and their professional values of care. This was enacted collectively when community nurses shifted their gaze outwardly from ‘technologies of the self’ to others on their team, demarcating appropriate practice for nurses more broadly through ‘technologies of the collective’. This paper contributes to governmentality studies by demonstrating the ongoing work required by professional communities to render themselves ethical within the modern neoliberal state.

## 1 Introduction

Self‐management for those with long‐term conditions has become central to recent international health policy, especially in developed countries (Douglas et al. 2020; Ellis et al. 2017). Underpinning these policies are discourses of empowerment and patient‐centred care, where patients become active agents harnessing their own knowledge and experience to take greater control and responsibility for their condition (Fox et al. 2005). However, health professionals, key mediators of such policies, must themselves undergo complex processes of subjectification (Jones 2018). Using a case study of a community nursing team in England tasked with implementing patient self‐management, this paper draws on Foucauldian theory of governmentality and pastoral power to explain the individual and social processes that take place when clinicians integrate self‐management within their professional identity. It highlights the crucial mediating role health professionals play in policy implementation and the nuanced ways agency and resistance are enacted when clinicians seek to demarcate the boundaries of appropriate practice for their professional community.

Foucault's (1991b) theory of governmentality refers to the form of power that emerged in the 18th century in response to the state's objectives to manage the problem of mass populations. Although the modern state gained responsibility for ensuring the welfare, wealth, longevity and health of the population, direct command and control no longer had practical expediency in achieving these goals. The modern state must instead direct the population using large‐scale campaigns or indirect techniques that act on the consciousness of individuals to achieve its broader aims. Governmentality goes beyond Foucault's earlier analysis of specific institutional forms of power (i.e., Foucault 1991a) to identify a wider perspective that sits behind institutions, which he refers to as a ‘technology of power’ (Foucault 2009, 116). To govern is to direct ‘the conduct’ of individuals and groups through ‘mentalities’ or ‘rationalities’ of government that delineate appropriate forms of behaviour and desirable subjectivities that align with central political objectives (Foucault 1991b; Gordon 1991).

Foucault (2009) traced the origins of this form of power back to the 16th century, where he describes the role of the Christian pastorate, using the metaphor of the ‘shepherd’ who guides his congregation or ‘flock’ into behaviours that will ensure salvation in the next life. This form of power is interested in both the collective behaviours of the flock while also being ‘individualising’ in its concern for individual members of the congregation or ‘sheep’ (127–128). For Foucault (1982), ‘pastoral power’ has been integrated into the modern state by those working as state officials, managers, health professionals and bureaucrats. These modern‐day pastors no longer seek to ensure salvation in the next life but attempt to shape the behaviours of individuals to meet broader population‐level objectives.

Policy drives for health professionals to work in partnership with patients and encourage responsibilised subjectivities, and so reduce the burden on the state, have proved a central case study in understanding governmentality (Wilson 2001). Evidence supports self‐management interventions that are tailored, flexible and offer a range of self‐management tools as leading to improvements in clinical outcomes (i.e., changes in blood pressure) and humanistic outcomes (health‐related quality of life) for patients across a range of long‐term conditions, such as asthma, COPD, diabetes and depression (Dineen‐Griffin et al. 2019). However, although acknowledging benefits arising from greater individual agency over health (Petrakaki et al. 2018), sociologists have sought to illuminate and critique the neoliberal economic rationality underpinning the empowerment discourse fuelled by reducing public expenditure on state provision (Mattioni et al. 2021).

Health professionals who act as modern‐day pastors to guide and shape patients into responsibilised subject positions (Atkin et al. 2021; Jones 2018; Waring and Latif 2018) have been identified as key intermediaries in translating governmental discourse in ways that are understandable and relatable to targets of power (Waring and Martin 2016). Healthcare intermediaries do not, however, present as fully formed pastors. The redefinition of professional roles (Atkin et al. 2021), as well as training programmes and managerial practices, have all aimed to constitute clinicians into pastoral subjectivities that seek to shape self‐management behaviours in patients (Jones 2018). Moreover, clinicians from different professional backgrounds who inhabit a plural discursive field may enact their pastoral role to responsibilise patients in competing ways (Waring and Latif 2018).

This paper seeks to build greater understanding of how health professionals are constituted as pastors through professional socialisation. It draws on ethnographic data collected between 2017 and 2019 from a community nursing team in England. During the period of public sector financial retrenchment in the United Kingdom (UK), known as the ‘decade of austerity’ (2010–2020) (Irving 2021), community nurses in this study were both encouraged to adopt pastoral subjectivities in their relationships with patients, while being intersubjectively related with nursing colleagues through supervisory relationships, sharing patient caseloads and inhabiting an open‐plan office space. This paper will draw upon and extend Waring and Martin's (2016) framework of pastoral practices by highlighting ‘practices of ethical negotiation’ as a key activity in collective professional socialisation. These practices took place when nurses struggled to reconcile sometimes competing discourses of ‘waste watching’ focused on identifying patients for discharge and their caring obligations to patients. These practices were enacted collectively when nurses moved their gaze outwardly from self‐regulation to demarcate boundaries of acceptable practice for the wider nursing community.

## 2 Governmentality, Pastoral Practices and Counter‐Conduct

Foucault's body of work sought to trace the establishment of dominant forms of knowledge (Foucault 2003), institutionalised power (Foucault 1991a) and the role of government in control of the population (Foucault 2009), often using health and medicine as a key example (McGivern et al. 2017). His concept, the ‘medical gaze’, explained how the ability of clinicians to observe, interpret and classify the body within hospitals led to the establishment of a dominant medical knowledge (Foucault 2003). Tracing the antecedent to more recent neoliberal health policy reforms focused on increasing individual responsibility, Foucault (1980) described a ‘medico‐administrative knowledge’ (176) emerging in the 18th century that sought to reconcile ensuring the health of society with limitations presented by administrative and financial constraints of the state. This included movements to replace hospitals with greater levels of care at home. In effect, his study of power moved from institutionalised forms of discipline to that which ensured the health and well‐being of the population through control of individuals at a distance (Gordon 1991).

Lectures delivered at the Collège de France between 1977 and 1980 set out Foucault's main argument for the ‘governmentalisation’ of the state in which whole populations are governed to ensure wealth, long life and health (Foucault 2009). Key to governmentality is the ability to affect the consciousness of individuals and to direct their conduct in such a way that they freely align their own self‐conception with central political objectives (Foucault 1982). These subjectivities are acquired through individualising work, referred to as ‘technologies of the self’, which encompass acts upon the body, conduct and ways of being that allow subjects to render themselves as ethical beings (Foucault 1988). Within the modern neoliberal state, ethical subjects are those who act upon themselves as free, entrepreneurial and competitive subjects, assuming responsibility for issues previously in the hands of governmental agencies (Burchell 1996).

Governmentality theory has been used to understand more recent reforms to healthcare organisation based on managed clinical networks, highlighting how the state can retain a strategic steering role in loosely managed networks of organisations and professions across geographic boundaries (Ferlie et al. 2012). By drawing on Foucault's concept of pastoral power, Waring and Martin (2016) sought to explain the role of clinical leads in coordinating clinical networks for patient safety in the English National Health Service (NHS). As ‘pastors’, leads are identified as ‘crucial nodes in neoliberalism’ (138), acting as the interface between clinical colleagues within their network and wider policy decision‐makers and stakeholders. It is pastors' ability to translate governmental rationalities in ways that are understandable and acceptable to their professional communities that Waring and Martin (2016) argue requires adequate theorisation of ‘technologies of the collective’. These practices explain how pastors work to collectively constitute the subjectivities of professional communities (Waring and Martin 2016).

Waring and Martin (2016) developed four pastoral practices to explain how leads, acting as modern‐day pastors, translated and communicated prevailing mentalities to colleagues within professional communities. Constructive practices involve leads receiving communications from local and national agencies that must be scanned, made sense of, relevant information selected and then translated into forms seen as legitimate among the professional community. Key here is aligning neoliberal and professional values by emphasising those practices that fit best with the values and norms of the professional community. Although constructive practices normally take place in private settings, inscription practices convey governmental discourse in public forums, such as weekly team meetings and training events. Through ‘describing and explaining’ the importance of initiatives during professional and organisational routines, these practices were described as having a ‘sermon‐like quality’ (142). Collective practices involve leads engaging in collective activities aimed at setting the boundaries and demarcating acceptable behaviour for the professional community. These usually occur in shared forums and meetings and involve openly discussing, reviewing and scrutinising colleagues' conduct. Finally, inspection practices represent ongoing monitoring, surveillance, and regulation by safety leaders to assure the conduct of the community.

Less is known about how pastoral practices operate among those at the ground level of governmentality. Although network leaders studied by Waring and Martin (2016) are described as having clear strategic and operational roles that link local networks and policy decision‐makers, those working in frontline care teams manage the majority of day‐to‐day healthcare delivery, and so play a key role in translating and enacting governmental rationalities to patients and each other. McGivern et al. (2017) discuss how network leaders' involvement in constructive practices helped them acquire expertise and legitimacy among their colleagues, adding to their pre‐existing professional and personal credibility. This ‘pastoral status’ enabled leads to better shape the subjectivity of colleagues within a clinical information network. Junior colleagues, who were encouraged to develop pastoral status themselves, created a ‘pastoral constellation’ where this role was distributed throughout the professional hierarchy. Although concepts of pastoral ‘status’ and ‘constellation’ deepen understanding of interpastoral relationships and the role of professionals beyond leadership, community nurses in this study were both encouraged to adopt pastoral relationships with patients while being intersubjectively connected with colleagues on their team, offering a novel and valuable empirical context in which to study pastoral relationships further.

The current framework also presents a level of harmony in how pastoral practices are enacted by leads at the collective level. Waring and Latif (2018), however, found that pastors inhabiting a plural discursive field may lead to competition over regimes of truth, resistance and agentic enactments of their pastoral role. For example, pastors may employ disciplinary means of surveillance and correction, while, at other times, gently shaping and guiding patients to adopt responsibilised subjectivities. Greater understanding of how competition, resistance and agency relate to collective pastoral practices is therefore needed to uncover the workings of power within a modern healthcare context.

Inherent within Foucault's (2009) understanding of pastoral power is the potential for resistance and ‘counter‐conduct’. Forms of ‘counter‐conduct’ to the 16th‐century Christian pastorate included asceticism, formation of communities, mysticism, return to scripture and eschatological beliefs. All forms of counter‐conduct involve rejection by the congregation of the pastor as the translator of God's word, with members instead stressing an individual and direct relationship between themselves and religious teachings. When making a ‘return to scripture’, the faithful will ‘short circuit’ the pastorate by returning to original texts, such as the Bible. This places them in the presence of God's word by allowing them to read texts themselves without the need for interpretation and rearticulation by the pastor. This individual reading of texts creates ‘inner illumination’ (213) through personal understanding of God's teachings.

The form ‘counter‐conduct’ takes in the practices of modern‐day pastors, who no longer lead people to religious salvation in the next world, but ensure individuals are shaped in line with meeting the health, well‐being and security of the population, has been less discussed (Foucault 1982; Waring and Latif 2018). Applied to this study, these forms of ‘counter‐conduct’ will help better account for how resistance and agency influence the collective constitution of professional boundaries for health professionals who are intersubjectively connected.

## 3 Case Study

This paper is drawn from a wider PhD study evaluating the implementation of integrated care policy at the local level (see also Kendrick and Mackenzie 2023). This study employed a qualitative case study research design and used ethnographic methods to collect data within a community‐based integrated care (CBIC) service within one region of England. The social enterprise, Oakley Community Care (OCC), was awarded the contract to deliver the service, which went live in 2016. Data collection took place between 2017 and 2019. Access to the lead provider organisation and staff was granted by senior management, who were involved in awarding the PhD studentship to evaluate the service change. The service was comprised of four co‐located integrated care teams, including community nurses and therapists and some hosted services, such as speech and language. Community nurses delivered nursing care, such as wound care, insulin administration and palliative care, to housebound patients in their homes. All place names, organisations and participants have been pseudonymised.

The CBIC was developed and implemented in the context of the UK Coalition and then Conservative government's policy of austerity between 2010 and 2020 (Irving 2021). During this period, the NHS budget was technically protected but did not meet rising demand and population increases (Robertson et al. 2017). In contrast, local authorities (local government covering individual English counties, responsible for services such as social care, schools and waste disposal) received a funding cut of 28.6%, severely curtailing spending on mental health and social care (Ferry et al. 2024). NHS England (2014), the arms‐length body overseeing the NHS, published the *Five Year Forward View*, outlining plans to save £20 billion by 2020 to address an ageing population, rising demand and financial constraints. The CBIC Business Case had the stated aim to align with this policy by integrating health and social care using integrated teams, care co‐ordination, person‐centred goal setting and self‐management. This paper draws on a subset of data from the PhD, collected from the community nursing team about the implementation of patient self‐management, within the political context of austerity.

## 4 Data Collection and Analysis

This paper was drawn from data collected through 3 weeks’ observation in two integrated care offices, 17 interviews with members of the nursing team and middle management, and analysis of three organisational texts. Texts selected for analysis were the PowerPoint presentation and training manual used during the health coaching training session and staff newsletter distributed to all staff during my time observing day‐to‐day working. Texts were selected as examples of communicative events that provided semiotic ‘points of entry’ (Fairclough 2008) into how discourse constructing self‐management was being framed and communicated to frontline staff.

The purpose of interviews was to capture staff experiences of organisational change efforts for increased self‐management and how this influenced identity shifts and interactions, and the role of contextual factors. Interviewees were selected purposively by professional role (see Table 1) and recruited via email or snowball sampling. Observations aimed to capture staff interactions and conversations in open‐plan integrated care offices, particularly discussions on promoting patient self‐management and its impact on care. During this time, I hotdesked in the office and recorded field notes on my laptop.

**TABLE 1:** Interviewees.

Name (pseudonym)	Role	Band	Description
Katherine	Integrated care manager	8	Hold significant management responsibilities for a large team or area.
Zoe	Nursing lead (management and clinical)	7	
Fiona	Nursing lead (management and clinical)	7	
Adele	Matron	6	Senior nurses who take on a leadership role in the team, while retaining clinical duties
Caroline	Matron	6	
Adrienne	Matron	6	
Lucy	Senior community nurse	6	
Emily	Senior community nurse	6	
Penny	Registered nurse	5	Professionally registered nurses with greater level of responsibility, including patient assessment and planning
Amelia	Registered nurse	5	
Raquel	Registered nurse	5	
Cheryl	Registered nurse	5	
Amber	Nursing assistant	3	Junior members of staff who deliver a wide range of clinical tasks to patients but do not have professional registration
Candice	Nursing assistant	3	
Phoebe	Nursing assistant	3	
Leah	Nursing assistant	3	
Sonia	Nursing assistant	3	

Organisational texts were analysed using critical discourse analysis semantic and linguistic analysis. Texts were analysed for metaphors used, ways in which responsibility is ascribed to agents, the expressive values attached to words and the expression of modality (degree of confidence in what is being said). These linguistic devices were analysed for how they drew on dominant narratives and discourses (Fairclough 2001). Interview and observation data were analysed separately from texts to capture enactment of roles and subject positions in response to the service change, although the purpose of textual analysis was to determine how self‐management discourses were realised linguistically. Initial coding involved assigning codes inductively to both interview and observation data to provide a conceptual label that explained what was being represented in the data at a higher degree of abstraction (Charmaz 2014). These concepts were categorised as contextual factors (individual, interpersonal and organisational factors that existed prior to the introduction of CBIC), resources (organisational and professional changes that were introduced to staff), responses (ways staff enacted their roles, interactions, ways of being and subject positions adopted as they responded to change) and outcomes (in terms of either patient or staff experience). I then wrote memos exploring how concepts related to one another and to discursive frames identified during textual analysis.

After initial coding and memoing, I drew on Foucault's (2009) theory of governmentality and pastoral practices (Foucault 2009), and more recent applications by Waring and Martin (2016) and Waring and Latif (2018), to link field observations with macro‐level theories of neoliberalism and staff's role in mediating organisational discourse. Here, I grouped initial codes into theoretical categories using Waring and Martin's (2016) framework of pastoral practices (i.e., constructive, inscription, collective and inspection), Waring and Latif's (2018) subjectifying and disciplinary pastors and Foucault's (2009) forms of pastoral counter‐conduct. For example, an empirical code ‘managing grey areas’ was then grouped under ‘collective practices’ and ‘return to scripture’.

During this stage, I also began grouping initial codes that were not explained by current theoretical concepts and writing memos to explore connections between the codes and to develop new conceptual understanding of what was being observed in the data. In the final stages of coding, I used these theoretically informed codes to conduct more focused coding on the remaining data (Charmaz 2014).

This study presented low risk to participants. The main ethical challenge was the potential for me to overhear confidential patient information without their consent during my observations. This was mitigated by only making anonymised and nonidentifiable field notes.

## 5 Findings

In the section below, I set out the two main discursive frames introduced to frontline staff via organisational communications.

### 5.1 Health Coaches as Subjectifying Pastors

Training sessions ran on an ongoing basis and were offered to all staff. The session I observed was delivered by a trainer using a PowerPoint presentation, group discussion/activities and a training manual. The introductory part of the training manual described health coaching delivered by clinicians as:

“working in partnership” to ….“empower them (the patient) to build up the knowledge, skills, confidence and resilience to be responsible for and manage their own health and well‐being and to access appropriate care and support as required”

Use of ‘working in partnership’ and ‘empower’ suggests a redistribution of power and reversal of the medical model that maintains a hierarchical relationship between patients and clinicians. However, this empowerment discourse (Bravo et al. 2015) is clearly specified around notions of activation and responsibilisation, where patients have ‘knowledge, skills and confidence’ to ‘be responsible for managing their own health’.

The training manual goes on to describe how this process of responsibilisation is achieved when clinicians, acting as ‘health coach’, use ‘health coaching conversations’ to ‘encourage the coachee to take ownership of their decisions’ and to ‘guide coachee through any experiences’. Attendees were also reminded by the trainer that self‐management is ‘not forcing things on people that they're not necessarily wanting’ and ‘respecting that person as an individual, as everyone's needs are different’. Here, clinicians were constructed as active mediators who use guiding, respectful and encouraging conversations to responsibilise patients. This suggests they are asked to adopt a subjectified form of pastoral power identified by Waring and Latif (2018), where health professionals encourage self‐reflection and gentle shaping of behaviours.

Nevertheless, on page 3 of the training manual we are told that the health coaching training has been developed as a ‘new approach to managing demand’ and is a ‘critical part of (OCC's) organisational strategy’. Financial demands are emphasised through a graph and accompanying text on page 4:

The (CBIC) contract value decreases over the lifetime of the contract. Cost improvement plans are in place, but we also need to reduce demand on our service through self‐management.

The graph titled ‘(CBIC) base contract value’ indicates the contract value falls by £1 million between 2017 and 2019. This stark financial message and graph visualisation work to emphasise to staff the underlying financial aims of adopting the role of health coach. Despite the construction of subjectifying pastoral roles enacting self‐reflection and individualised guidance, there is clearly an instrumental aim underpinning these activities to reduce cost pressure (Petrakaki et al. 2018). This might suggest that within an empowerment framing, health professionals perform a pastoral role by negotiating between individualised patient goals and financial requirements.

### 5.2 ’Waste Watchers’ as Disciplinary Pastors

During the period of the £1 million contract reduction highlighted above, senior management became more focused on reducing costs. In 2018, OCC senior management launched the ‘Transformation projects’, which were communicated to staff through the September 2018 staff newsletter and discussed during staff meetings. A suggestion box and noticeboards were located in offices for staff to add ideas.

The staff newsletter set out the need to remove ‘waste’ from care delivery to achieve cost savings:

Removing or reducing waste – be it wasted intellect, surplus stock or excess travel – can all have a positive impact on our overall organisational cost savings.

The newsletter then described how this will be achieved through staff ‘becoming a “Waste Watcher”’ themselves and the establishment of a ‘Transformation Team’ and ‘transformation projects’.

The Transformation Team are currently supporting 5 transformation projects which have been developed directly from feedback provided by staff who attended the Service Redesign Workshops on 30th and 31st August. The philosophy of waste reduction runs through each of these projects.

The ‘transformation projects’ included 1. Facilitated Health and Well‐being, 2. Model Team, 3. Agile Working, 4. Effective Technology and 5. Managing Supplies. Self‐management was framed through a ‘waste watching’ narrative in the Facilitated Health and Well‐being project, as expressed in the following statement:

Uses ‘**waste watchers**’ philosophy; reducing the time that patients are in [OCC’s] services by giving them the necessary tools to self‐manage their care. Facilitated Health and Wellbeing also aims to enhance patient care by giving patients the tools they need to better manage their health and wellbeing.

Here, the position of clinician and patient is constructed differently from within the empowerment discourse. Firstly, use of the word ‘waste’ has potentially negative connotations for patients through association with something that needs disposing of. Although naming staff as ‘watchers’ draws an association to monitoring and surveillance undertaken by disciplinary pastors (Waring and Latif 2018) who seek to identify potential ‘waste’ that can be removed. The claim this will ‘enhance patient care’ suggests that, similarly to health coaching, ‘waste watching’ will simultaneously improve care and achieve cost savings. However, replacing working in partnership, empowering patients, and treating them as individuals with ‘giving patients the tools they need to better manage their health and wellbeing’ suggests authority remains with clinicians and constructs the pastoral role as responsibilising patients through disciplinary and hierarchical means.

### 5.3 Constructive, Inscription and Collective Practices: Recoding and Enacting ‘Waste Watching’ in the Everyday Lives of Community Nurses

#### 5.3.1 Constructive Practices

- Translating and recoding discourse

Nurse interviewees reported they had not participated or struggled to clearly articulate the purpose of the health coaching training, and therefore, little emphasis was given in interviews to achieving patient empowerment through self‐management. Instead, nurses talked of the need to ‘get people off the books’, which meant discharging patients from the service and freeing up capacity. This seemed to closely reflect ‘waste watching’ assumptions where patients wasting ‘intellect’ and ‘travel’ could be identified and discharged. However, getting ‘people off the books’ could be seen as recoding managerial discourse into language that was acceptable, relatable and easily understood by members of the team, indicative of ‘constructive practices’ (Waring and Martin 2016).

In a further example of ‘constructive practices’, community nurses sometimes spoke interchangeably of discharging through self‐management and under the housebound criteria. Eligibility criteria for community nursing included those who cannot leave home or require substantial assistance to leave due to health issues. Those not meeting these criteria can be discharged. On occasion, I would ask interviewees about self‐management, and they would respond by talking about the housebound criteria. This was highlighted when I asked senior nurse Lucy to provide an example of a self‐management success.

I think the thing is, it's not even a success, is it? Most of them that you'll find are not housebound. So, the success is you've discharged them back to the appropriate service.

Lack of salience of health coaching in the everyday working lives of nurses meant an absence of empowerment and goal setting that may have worked to distinguish discharge through self‐management from housebound criteria. Instead, recoded forms of ‘waste watching’ come to encapsulate both types of patient–clinician interactions as potential opportunities for getting patients ‘off the books’.

Although there was no evidence that senior nurses used private settings to consciously scan and translate managerial discourse of ‘waste watching’ into a form acceptable to the wider professional community, fieldwork suggested they performed an important role in recoding and communicating the need to get patients ‘off the books’ to the wider team. Senior community nurses and matrons were responsible for supervising more junior staff and providing clinical leadership. Their responsibility in the team for patient triage also meant they were often physically present in the office and appeared well liked and respected by junior colleagues, who were observed and self‐reported frequently consulting senior nurses for patient advice. Senior nurses also attended the transformation workshops (discussed above) alongside the senior management and so were directly involved in organisational planning to operationalise ‘waste watching’. This influential role in mediating managerial discourse of ‘waste watching’ therefore afforded them with the expert, professional and personal credibility that provided ‘pastoral status’ (McGivern et al. 2017) among their professional community.

b. Aligning governmental discourse with professional values and identity

Nurses at all grades, however, described an ongoing process of making sense of how ‘waste watching’ aligned with their professional roles. This often involved enacting their pastoral role flexibly in response to a heterogeneous patient population. Nurses described ‘running around like no tomorrow’ and ‘firefighting because we haven't got no capacity’, which meant that for clear‐cut cases they found easy alignment between their professional values and ‘waste watching’. Descriptions were given in interviews of ‘catching people out’ or ‘finding out’ that people were not housebound or, in their opinion, did not have the capacity to deliver their own care. In these cases, ‘waste watching’ was viewed as aligning with the norms of their professional community, as it did not contravene their caring obligations to patients. For example, registered nurse Penny told me, ‘Well, actually no, it's not my job’, in response to a resistant diabetic patient she deemed capable of taking responsibility for his insulin injections.

Nurses also described when patient vulnerability meant maintaining a more traditional caring role. These patients were primarily housebound, elderly and exhibited poor mobility, eyesight and dexterity that rendered insulin administration difficult. Registered nurse Raquel said of these patients, ‘they've actually proven that they can't do it because they've had hypos, hypers, ended up in hospital, they've been found on the floor’. However, other scenarios were described that required a greater level of ethical negotiation when attempting to reconcile what nurses saw as competing discourses of waste watching and their professional values of care, as described below by nursing assistant Sonia.

I just feel a great deal of the patients that we see will never be able to self‐manage, you know. I know there's a big thing about housebound at the moment. Housebound patient, you know “Tell me what is a housebound patient?” I find it quite difficult and I'm not the toughest on that sort of thing, but basically if they can get in a car, they're able to get to the surgery by taxi whether they've got the money or not because it's not our concern. I do struggle with that a bit.

Her self‐questioning, ‘Tell me what is a housebound patient?’, implies she discerns ambiguous criteria that must be grappled with when making applicability decisions. Sonia's reference to not showing concern for the cost of a taxi suggested she viewed her pastoral expectations to apply housebound criteria without consideration of broader issues as both overly simplistic and reneging on her obligations to patients. She described these decisions as ‘difficult’ and a ‘struggle’, indicating a level of ethical conflict between the constructive practices of her pastoral community and her personal views on who should be eligible for care. By being ‘not the toughest on that sort of thing’, Sonia suggested she regulates her own professional behaviour through ‘technologies of the self’ in line with these personal convictions.

Similarly, matron Adrienne conveyed her unease when she described below the ‘little someone in your ear saying you've got to discharge all these patients’, suggesting an internalised form of organisational discipline and surveillance that contravenes her judgement and makes her uncomfortable when interacting with patients.

I just think there's sometimes that pull of, like that little someone in your ear saying you've got to discharge all these patients, and they’ve all got to be self‐caring. And sometimes that is lost because you just think, I mean I’ve been out to a couple of patients and I think you're pushing them to… These patients are so sad and so stressed because they're being told they've got to take over this skill, and they're just not capable of doing it.

Similar to Sonia, Adrienne expressed disagreement with how members of her professional community are aligning ‘waste watching’ with their professional duties of care, accusing them of ‘pushing’ patients to become ‘sad’ and ‘stressed’. In effect, Adrienne and Sonia made a ‘return to scripture’ where they return to their own judgements formed through a direct relationship with patients. The ‘inner illumination’ this created (Foucault 2009) allowed them to regulate their professional conduct by seeking to realign the balance between governmental discourse and professional values constructed by their professional community to one that reflected their own ethical understanding of the nursing pastoral role. These findings suggest our understanding of constructive practices needs to encompass these ‘practices of ethical negotiation’, which reflect feelings of discomfort and difficulty towards interpretations of the wider professional community, leading pastors to undergo a process of realigning their pastoral role to be compatible with personal convictions.

#### 5.3.2 Inscription Practices

Senior nurses spoke about how they used their position within the team to encourage staff to get patients ‘off the books’, while also acknowledging the potential difficulties of alignment with professional values held by nurses in the team.

If you’re a trained nurse or in the caring profession, you want to look after people and you want to look after them until you’re 100% sure they're okay. And it's that letting go bit that can cause an issue. But they're getting better and that's because we're driving it. Really trying to drive it by saying to them, “We can get these things off the books. You know, your caseloads are going to be lighter for a while, if you’re stricter with things that come in.”

In the above quote, senior community nurse Emily described ‘really trying to drive’ nurses in her team to discharge patients to self‐management. Part of this drive involved encouraging nursing pastoral subjectivities that can let go of some of their caring role to ‘look after them until you're 100% sure they're okay’ and instead be *‘stricter’* when assessing patient eligibility for the service.

Nursing lead Zoe also explained how she communicated identifying and discharging nonhousebound patients to her staff.

I explained it to my staff in the last team meeting. You don't go into a shop and pick up your shopping and pay for half and walk out with the other. You only get what you've paid for. For community nursing and under (CBIC), we're only paid to do X,Y and Z. We're only paid within our contract to see housebound patients. If we're seeing patients that are going out, which we do at times, and find out. We need to have those conversations. That's really hard from a caring professional nurse.

In this example, Zoe used her managerial position leading the team meeting to explain the importance of the change and rendered it understandable to staff through her shopping analogy. Expectations of appropriate pastoral behaviour were indicated by telling nurses that if they ‘find out’ that patients are leaving their home, then they ‘need to have those conversations’. Similarly to Emily, her reference to this being ‘really hard from a caring professional nurse’ is an acknowledgement that nurses must integrate a new set of rationalities within their existing professional identity and that this may create ethical tensions for some.

Emily and Zoe's tactics suggest the use of ‘inscription practices’ to shape staff's subjectivities. Both employed forms of ‘describing and explaining’ the importance of the change, legitimising and encouraging them to internalise these norms into their professional practice (Waring and Martin 2016, 142). Their acknowledgement of the need to integrate subjectivities associated with getting patients ‘off the books’ and those of their caring profession also reflected ‘collective practices’ that redraw boundaries of acceptable nursing practice (Waring and Martin 2016).

#### 5.3.3 Collective Practices

Co‐location in the open‐plan office and team meetings provided forums where to ‘get people off the books’ could be collectively reinforced. Nurses described frequent subtle reminders or suggestions within the open‐plan office that sought to provide this collective reinforcement. Below, registered nurse Cheryl described ‘a little nudge’ from senior nurses:

Yes, every now and again, there'll be a little nudge. You walk into the office and it will be like, you know whoever is walking into the office at that time it will be, “Today, we're going to get some patients off”, for something.

Cheryl's quote highlights the role senior nurses played in reminding staff in the office to ‘get some patients off’. However, nurses at all levels were observed in collective reinforcement of these behaviours. During observations within the office, I heard a registered nurse say to a new starter, ‘we have to try to encourage patients to take more care of themselves’. I also heard the question ‘are they housebound?’ being asked between nurses on numerous occasions. This dissipation of practices reinforcing pastoral subjectivities among the wider team could indicate further ‘technologies of the collective’ and intersubjective socialisation that demarcated collective boundaries through subtle reminders and questions from colleagues. Nurses in this study were, therefore, expected to enact pastoral roles in their relationships with patients, while also being members of a pastoral community that influenced each other through collective practices.

There was evidence, however, that ‘practices of ethical negotiation’, where nurses struggled with the balance between ‘waste watching’ and professional values of care adopted by colleagues, were reinforced collectively. As a senior nurse, Adrienne described how her ‘return to scripture’ took on a collective dimension when she sought to reaffirm acceptable boundaries of nursing practice as holistic and orientated around the patient.

Sometimes that part is lost where the more holistic side of it, I suppose. Some are just really task‐orientated. I think that's a shame if that's— I don't want nursing to go that way of all being around the task. It's got to still remain around the patient.

In another part of her interview, she expressed concern that some junior members of staff were unilaterally enforcing the housebound criteria and would ‘just discharge without telling the lead nurse, or anything’. In these cases, she said she tries to advise staff that ‘it's a guideline’ and ‘we need to use our initiative’ to prevent harm to patients. She therefore draws on her ‘pastoral status’ to encourage pastors to flexibly negotiate nursing values and ‘waste watching’.

In another example, I observed the following interaction in the office, which involved open securitising of the work of nursing colleagues, reflective of a collective practice.

Kath (senior community nurse) says to Cheryl (Registered Nurse) ‘*I mean she does have some fair points*’. The patient is being treated for leg wounds by the service but is also going to the hospital once a week to have blood transfusions and these are keeping her alive. Kath goes on to explain ‘*she’s a palliative patient*’. Kath tells Cheryl that when the nurses hear about this ‘*they rightly say, well you’re not housebound then*’. Kath urges ‘*We need to have some flexibility with these kinds of patients. We need to be treating them on an individual basis*. *So, I’ve said to her that we’ll still go out and see her as she’s really poorly and palliative*.’ Cheryl replies ‘*people are just applying it so rigidly to everyone and we need to treat people individually’*. Kath replies ‘*I know, they need to be treating each patient at a time. I mean especially if they’re palliative, I mean they’re going through enough as it is. We just need to make sure that no one else says to the daughter that her mum’s not housebound. I think one more time and it’ll tip her over the edge’*.

Kath criticises nursing colleagues who ‘need to have some flexibility’ with patients by ‘treating them on an individual basis’. Kath's reference to nurses ‘rightly’ identifying the patient as housebound suggests she agrees in principle with this form of waste watching. However, similarly to Sonia and Adrienne, it was first‐hand observation of patient need as ‘poorly and palliative’ and negative impacts on the patient’s daughter, which triggered negotiation of the ethical demands of her pastoral role by reinstating care. This was reinforced collectively when Kath said, ‘We just need to make sure that no one else says to the daughter that her mum’s not housebound’. This demonstrated the important role played by senior nurses in the ongoing work of ascribing appropriate behaviour within a pastoral community.

The ongoing role played by senior nurses in redefining appropriate professional practice through ‘grey areas’ was also discussed by senior community nurse, Emily.

So there are very many grey areas and they’re often the ones that we’re left sorting. We kind of just discuss it amongst ourselves really and if we’re not sure, we’ll ask Zoe (Nursing Lead). She will say, “Discharge” and offer our services if they ever do become housebound, then tell them to call in again. But we tend to usually come to a bit of a compromise, to ease them out a little bit. Otherwise, you’re just going to get a massive complaint coming in which takes up hours of someone’s time to do it.

The collective dimension to working out how best to enact their pastoral role is highlighted when Emily says they ‘discuss it amongst themselves’. Interestingly, although they consult a manager with greater seniority for difficult cases, Zoe’s more clear‐cut advice to ‘Discharge’ is again reinterpreted when senior community nurses ‘come to a bit of a compromise to ease them out a little bit.’ Here, senior nurses deem themselves to have greater first‐hand knowledge and ‘inner‐illumination’ of the impact on the team at large, that is, taking up time when dealing with the inevitable complaint, and so ‘short circuit’ (Foucault 2009) Zoe.

## 6 Discussion/Conclusion

This paper draws on data collected from a case study community nursing team in England delivering NHS services, who acted as pastors tasked with shaping patients into responsibilised subjectivities while being members of an intersubjectively related pastoral community. This paper draws upon and expands Waring and Martin's (2016) model of pastoral practices in three main ways. Firstly, the concept of constructive practices is broadened to encompass translating and recoding practices that do not necessarily involve strategic and purposeful translation activities within a private setting. Findings presented here demonstrate instead how constructive practices can involve recoding discourse using existing linguistic devices (i.e., ‘getting people off the books’), as well as encompassing a broader range of practices within a particular rationality (i.e., encapsulating self‐management and housebound criteria within ‘waste watching’), in ways that make sense to the professional community (Mcnamara 2010), Similarly to constructive practices described by Waring and Martin (2016), these forms of recoding work effectively because they are easily communicated and have legitimacy. However, they differ by occurring spontaneously in response to challenges faced by the professional community, as opposed to intentional strategies designed to persuade.

Where patients had capacity to self‐manage, or clear patient vulnerability justified intervention, nurse pastors could be seen as ‘making sense’, ‘selecting’ and ‘translating’ (Waring and Martin 2016, 141) ‘waste watching’ into their professional practice flexibly and with little conflict. However, constructive practices are elaborated in this paper to encompass ethical deliberation when pastors disagree with interpretations made by those in their pastoral community that could potentially cause patient harm (Wong et al. 2024). These ‘practices of ethical negotiation’ manifested in *personal questioning*, *finding it difficult*, *struggling* and *feeling uncomfortable* and reflected findings from existing studies about nurses' ethical dilemmas when balancing care with potential harm and workload pressures (Haahr et al. 2019). By offering a conceptualisation of the ethical dimension of constructive practices, this paper demonstrates the difficult and ethically fraught nature of finding alignment between governmental discourse and professional values of care.

Lastly, this paper elaborates Waring and Martin's (2016) framework by conceptualising ‘practices of ethical negotiation’ as a collective practice. Nurse pastors regulated themselves through ‘technologies of the self’, coming to different judgements about the appropriateness of self‐management and housebound criteria, dependent on patient circumstances and practical impacts on colleagues. Collective‐level enactment included advising colleagues to adopt a more flexible approach, overruling colleagues' decisions and ‘short circuiting’ their superiors to come to compromises themselves. Clinicians who are themselves both pastors and members of the congregation are interested in their own ethical regulation before shifting their gaze outwardly from ‘technologies of the self’ to the rest of the flock, demarcating appropriate practice for nurses more broadly through ‘technologies of the collective’. Practices of ethical negotiation provide a more complete account of how pastoral power, which is both interested in the collective behaviour of the flock while also being deeply individualising (Foucault 2009), plays out within professional communities.

This paper builds on previous studies of collective pastoral practices within the context of healthcare networks (i.e., McGivern et al. 2017; Waring and Martin 2016) to deepen understanding of how pastoral communities operate within health professional teams. Reflecting ‘pastoral constellations’ (McGivern et al. 2017), community nurses of all grades were found to be deeply embedded in constructive practices, whereas senior nurses who had personal and professional credibility used their ‘pastoral status’ to guide colleagues' conduct. However, the ‘constellation’ concept may be more reflective of pastors connected through loosely managed networks, whereas the community nursing team in this study acquired deep intersubjective relationships through sharing of caseloads and frequent interaction in the open‐plan office. This proximity enabled more distributed, iterative and co‐constructed forms of collective practices, such as interprofessional reminders and questions and short‐circuiting superiors.

Elaboration of the framework presented here advances understanding of how agency and resistance are embedded within collective professional socialisation by more thoroughly incorporating Foucault's (2009) work on counter‐conduct into the model of pastoral practices. Similarly to resistance to the 16th‐century pastorate (Foucault 2009), when the faithful struggle with reconciling their own personal connection to God with the pastor's mediating role, modern‐day pastors wrestled with colleagues' interpretations versus their own responsibilities to patients. ‘Inner‐illumination’ is not obtained through a direct connection with God, but with first‐hand observations of impact on patients or those affected by governmental rationalities. Instead of returning to holy texts or ‘scripture’ for an unmediated connection with God, modern‐day pastors observe and judge the impact on patients and return to their own conception of appropriate professional behaviour. Finally, ‘short circuiting’ the pastorate may involve small acts of resistance or compromise between personal beliefs and directives from superiors.

Findings in this study did not, however, reveal what Foucault (2009) described as ‘formation of communities’ (p. 208) on moral grounds in opposition to pastoral authority. For example, community nurses might have formed a professional community to enact collective practices that rejected ‘waste watching’. Jones (2018) argues that efforts to enrol and train health professionals in promoting self‐management often fail due to entrenched identities. However, this study suggests that development of an ethical professional community around self‐management may require ongoing ethical work and negotiation, as clinicians interact with patients, family members and each other. It is for this reason that ‘practices of ethical negotiation’ are not simply ‘counter‐conducts’ that aim to ‘discredit pastoral power by promoting new obligations and morality’ (Foucault 2009, 204). Rather, they are continuous efforts to reconcile competing discourses to render both the self and their community ethical. The ongoing nature of ethical negotiation may also reflect why inspection practices, which involve disciplinary forms of audit and evaluation based on more definitive assessments of professional behaviour, did not feature prominently in the data.

Findings presented here have implications for policy and practice, given the ongoing global focus on managing chronic conditions through self‐management interventions (Li et al. 2025). The World Health Organisation recommends implementing self‐care strategies across all countries and economic settings to promote health and well‐being (World Health Organization 2022). Poor performance of health services, funding and workforce shortages across OECD countries (OECD 2023) and the Global South (Jakovljevic et al. 2024) highlight challenges to implementing self‐management under conditions of resource constraint, where, as this paper finds, frontline staff are often overwhelmed by reducing caseloads. Self‐management interventions may therefore increase chances of moral injury experienced by frontline staff who take on the burden of reconciling systemic tensions within neoliberal healthcare systems.

Although evidence supports multicomponent, long‐term strategies that help patients problem‐solve and work towards meaningful goals (Dineen‐Griffin et al. 2019), implementation in this study diverged from the more subjectifying health coaching approaches promoted in training. A ‘waste watching’ approach was found to be applied flexibly in clear‐cut cases where patients could safely manage, helping to reduce pressure on community nurses. However, disciplinary enactments of the pastoral role have the potential to neglect patient care needs and generate conflict within professional communities.

The argument advanced in this article is based on a relatively small case study in England and so may lack transferability to other international contexts. For example, how health professionals construct their identities in relation to technologies of the self and the community may differ across cultural and financial contexts. The claim made here that community nurses engaged in ongoing work to render themselves and their professional community ethical is predicated on having sufficient space to move between competing discourses to manage grey areas, even under resource constraints. Although concerns expressed by participants may reflect dangers of efficiency hindering professionals' ability to show empathy and holistic care (Kerasidou 2019), pastoral ‘practices of ethical negotiation’ may have worked to limit harmful effects of austerity policies on patients (Stuckler et al. 2017). Health workers in low‐ and middle‐income countries may find constituting ethical identities difficult in such highly resource‐constrained environments (McGivern 2025), compared to wealthier nations like the United Kingdom (OECD 2023).

Pastors in other contexts may also negotiate radically different discursive fields than those in Western secular societies. For example, health workers in Papua New Guinea were found to be drawing together and negotiating Christian and medical knowledge in delivering HIV prevention services (Shih et al. 2017). Further research is therefore needed to examine the relevance of theoretical propositions advanced in this paper in different national contexts.

## Author Contributions

**Hannah Kendrick:** conceptualisation, data curation, formal analysis, investigation, methodology, project administration, writing – original draft, writing – review and editing.

## Ethics Statement

Ethical approval was gained for this study from the East Midlands—Leicester Central Research Ethics Committee on 26th March 2018, ref: 18/EM/0084.

## Consent

The author have nothing to report.

## Conflicts of Interest

The author declares no conflicts of interest.

## Permission to Reproduce Material From Other Sources

The authors have nothing to report.

## Data Availability

Consent was obtained to use documents, interview transcripts and observation notes in reports and publications but not to make full transcripts, internal documents and notes available. Public availability of all data would contravene patient confidentiality and risk making participants and organisations identifiable within the research.
